# Gold decorated bismuth sulfide nanorods for enhanced computed tomography imaging with in vitro and in vivo validation

**DOI:** 10.1186/s11671-026-04708-1

**Published:** 2026-06-10

**Authors:** Bhavesh D. Kevadiya, Rosita Primavera, Ganesh Swaminathan, Jim Zhong, Anandakumar Sarella, Rajendra Prasad, Bela Desai, Jing Wang, Kenneth S. Hettie, Paolo Decuzzi, Avnesh S. Thakor

**Affiliations:** 1 Department of Radiology, Interventional Radiology Innovation at Stanford (IRIS), Stanford University, Palo Alto, CA 94304 USA; 2 Department of Diagnostic and Interventional Radiology, Leeds Teaching Hospitals NHS Trust, Leeds, UK; 3 Nebraska Center for Materials and Nanoscience, University of Nebraska Lincoln, Lincoln, NE 68588 USA; 4 School of Biochemical Engineering, Indian Institute of Technology (BHU) Varanasi, Varanasi, 221005 U.P. India; 5 Molecular Imaging Program at Stanford (MIPS), Department of Radiology, Department of Otolaryngology-Head and Neck Surgery, Stanford University, Stanford, CA 94305 USA; 6 Division of Oncology, Department of Medicine, Stanford University School of Medicine, 269 Campus Drive, Stanford, CA 94305 USA; 7 Laboratory of Nanotechnology for Precision Medicine, Fondazione Istituto Italiano di Tecnologia, Via Morego 30, 16163 Genoa, Italy

**Keywords:** CT imaging, Gold nanoparticles, Bismuth sulfide nanorods

## Abstract

The development of hybrid metal nanoparticles (NPs) for use as computed tomography (CT) contrast agents is a promising area of research. Achieving optimal in vivo performance of imaging for NPs is challenging and depends on their geometry, materials properties, bioreactivity, and biocompatibility. In this study, we designed and developed a novel CT contrast agent composed of gold nanoparticles (AuNPs) coordinated on the surface of bismuth sulfide-core nanorods (Au@Bi_2_S_3_ NRs) utilizing a solvothermal synthesis approach. We conducted solid-state characterization of Au@Bi_2_S_3_ NRs, demonstrating their structural configuration, excellent stability, uniformity, and high crystallinity. We also tested their biocompatibility with mesenchymal stem cells and found that they were well tolerated at lower tested concentrations, with reduced viability observed at higher concentrations. To evaluate the imaging potential of Au@Bi_2_S_3_ NRs, we tested them in small animals using CT imaging. Our results showed contrast enhancement in soft tissues, indicating the retention of the particles at these locations with no local inflammatory responses. Taken together, our study provides a proof-of-concept for the robust synthesis and use of Au@Bi_2_S_3_ NRs as effective CT imaging contrast agents. Future work will explore the potential to functionalize Au@Bi_2_S_3_ NRs with therapeutic molecules for theranostic applications.

**Supplementary Information:**

The online version contains supplementary material available at 10.1186/s11671-026-04708-1.

## Introduction

Metal nanoparticles (NPs) have unique properties that make them ideal for bioimaging applications, but their potential toxicity must be carefully considered [1, 2]. The high-energy blocking capabilities of metal NPs make them useful for computed tomography (CT) imaging [3, 4]. CT is a widely employed imaging modality that utilizes X-rays to generate detailed three-dimensional images of the body, relying on the varying densities of tissues to block X-rays to different extents [5–7]. Despite its effectiveness, distinguishing between tissues with similar densities can be challenging due to subtle differences in X-ray absorption, which may complicate image interpretation. To address this issue, contrast agents are employed. These agents can either accumulate in specific tissues or fill luminal spaces, such as blood vessels or the gastrointestinal tract, thereby enhancing X-ray absorption and improving image contrast [8]. Commonly used agents, like iodine and barium, can increase the radiodensity of tissues within which they reside, and are generally considered safe [9, 10], though they can be associated with adverse reactions, thyroid gland dysfunction, and nephropathy [11–13]. Additionally, the transit of these conventional contrast agents through tissues is relatively rapid, especially when administered via a vascular route; accordingly, precise timing is required for imaging or repeated CT dosing. However, these issues can potentially be improved by using electron-dense nanomaterials that can be retained longer in tissues [14–16].

In recent years, high–atomic number metal nanoparticles (NPs) have been increasingly investigated as next-generation CT contrast agents because they can offer improved biodistribution, biostability, predictable cellular interactions, and reproducible synthesis compared with conventional iodinated agents [1, 2, 4, 17–19]. A range of inorganic nanomaterials, including gold- and bismuth-based systems [4, 15, 20] as well as tantalum oxide and tungsten oxide [21, 22], have demonstrated strong X-ray attenuation, favorable biocompatibility, and in some cases, prolonged vascular retention in preclinical models. Among these, sphere-shaped gold nanoparticles (AuNPs) are attractive due to their high X-ray attenuation, low toxicity, facile synthesis, and straightforward surface functionalization for colloidal stability [15, 23–27], while rod-shaped bismuth sulfide nanorods (Bi_2_S_3_ NRs) provide strong X-ray absorption, excellent biocompatibility, high stability, and relatively long circulation times [4, 20, 28–30]. Collectively, these advances motivate the development of hybrid architectures (e.g. Au@Bi_2_S_3_ NRs) designed to integrate complementary material properties within a single platform and potentially improve imaging performance beyond that of either component alone.

While Bi_2_S_3_ NRs provide strong attenuation due to bismuth’s high atomic number, incorporating AuNPs into a Au@Bi_2_S_3_ hybrid architecture can provide additional advantages beyond attenuation alone. Gold possesses a K-edge at 80.7 keV, which complements the bismuth K-edge at 90.5 keV and lies within the diagnostic CT energy range, thereby enabling broader spectral attenuation and the potential for dual-energy or spectral CT applications [31, 32]. Furthermore, gold provides a versatile surface chemistry platform through well-established Au–thiol interactions, which affords the conjugation of targeting ligands, biomolecules, and therapeutic agents for future theranostic applications. The presence of gold also contributes to its apparent enhanced physicochemical stability by partially passivating the Bi_2_S_3_ surface, which is otherwise susceptible to oxidation under ambient conditions when gold is not in bulk form. In addition, gold nanoparticles exhibit surface plasmon resonance in the near-infrared region, offering the potential for photothermal therapeutic functionality (e.g., ablation). Together, these features make the Au@Bi_2_S_3_ hybrid system a multifunctional platform that allows for integrating imaging performance with chemical versatility and translational potential. These considerations motivate robust, reproducible synthetic strategies that yield stable, water-dispersible Au@Bi_2_S_3_ NRs suitable for biological evaluation.

In the present study, we developed an approach to reproducibly make a hybrid metal gold-bismuth-sulfide particle (i.e., Au@Bi_2_S_3_ NRs) of high quality, uniformity, and stability using a solvothermal synthesis approach. Here, spherical AuNPs were decorated onto the surface of Bi_2_S_3_ NRs in situ whereby the resulting Au@Bi_2_S_3_ NRs were bioanalytically characterized using state-of-the-art tools, followed by *in vitro* and in vivo biocompatibility testing and in vivo imaging using CT. The synthesis approach presented in this study differs from previously reported methods in several important aspects. First, a lipid-templated strategy using DSPE-PEG_2000_ is employed during the AuNP seeding process, which simultaneously stabilizes the Bi_2_S_3_ NR surface, provides a biocompatible PEGylated coating, and enables direct incorporation of functional lipid derivatives without requiring additional post-synthesis modification steps. Second, the synthesis is performed using a one-pot aqueous-phase process, which both eliminates the need for multiple solvent exchange steps and improves their reproducibility. Third, the use of controlled reduction conditions allows for the formation of uniformly distributed ultra-small AuNPs of tunable size and high surface coverage on the Bi_2_S_3_ NRs. Finally, the use of bismuth neodecanoate as a precursor results in highly crystalline Bi_2_S_3_ cores with low size variability. Collectively, these features provide improved control over particle structure, surface chemistry, and batch-to-batch consistency compared with existing methods and position Au@Bi_2_S_3_ NRs as a rationally-designed hybrid platform for CT contrast enhancement with potential for future multimodal imaging and theranostic extension.

## Experimental section

### Chemicals and reagents

Gold (III) chloride trihydrate (HAuCl_4_·3H_2_O), thioacetamide (CH_3_CSNH_2_), bismuth neodecanoate (Bi(OCOC(CH_3_)_2_(CH_2_)_5_CH_3_)_3_), sodium borohydride (NaBH_4_), oleic acid (OA), oleylamine (OAm, 70%), and L-α-phosphatidylcholine (PC) (from egg yolk), were obtained from MilliporeSigma, St. Louis, MO, USA. 1,2-distearoyl-sn-glycero-3-phosphoethanolamine-N-[amino(polyethylene glycol)-2000-fluorescein (sodium salt) (FITC-DSPE-PEG_2000_) was obtained from Xi’an ruixi Biological Technology Co, China. DSPE-PEG_2000_ (N-(carbonyl-methoxypolyethylene glycol 2000)-1,2-distearoyl-sn-glycero-3-phosphoethanolamine, sodium salt) was obtained from NOF Corporation, Tokyo, Japan. Human Wharton’s Jelly-derived mesenchymal stem cells (UC-MSCs) were obtained from StemBioSys, San Antonio, TX, USA. DAPI (4’,6-diamidino-2-phenylindole) was obtained from Thermo Fisher Scientific, USA. Cell culture media MesenCult™ and human platelet lysate were obtained from Stemcell Technologies, Vancouver, BC, CA. CellTiter-Glo™ was purchased from Promega, Madison, WI, USA. Male rats were purchased from Charles River Laboratories, USA.

### Synthesis of Au@Bi_2_S_3_ NRs

Core bismuth sulfide (Bi_2_S_3_) nanorods (NRs) were synthesized using the solvothermal method [29] followed by decoration with AuNPs. Briefly, 1.45 g (~ 1.87 mmol) of bismuth neodecanoate and 20 mL of OA were placed into a 500 mL capacity Teflon-lined autoclave reactor and the mixture was stirred for 10 min. Subsequently, 10 mL of ethanol was added dropwise and mixed for 10 min at 300 rpm. In a separate glass vial, 150 mg of thioacetamide (~1.99 mmol) was mixed with 4 mL of OAm and sonicated until a uniform, light yellow-colored solution was obtained. The thioacetamide-OAm mixture was then transferred to a Teflon-lined stainless-steel autoclave, and the solution turned black-colored. This mixture was stirred for 1 h, placed in a sealed autoclave that was maintained at 150 °C for 8 h, and then allowed to cool to room temperature. The precipitate was collected by centrifugation, washed several times with 200-proof ethanol, and dried overnight in a desiccator. The Bi_2_S_3_ NRs were finally obtained as a black powder. The Bi_2_S_3_ NRs were decorated with AuNPs using a seed-mediated growth method, which involved the following steps: Step one—Bi_2_S_3_ NRs (25 mg) powder was dispersed in anhydrous chloroform (1 mL). DSPE-PEG_2000_ (25 mg) was mixed in chloroform (1 mL) in a separate glass vial. Both components were bath sonicated to ensure they were completely dispersed and dissolved. Step two—the solutions were mixed with 2 mL of DI water, and the solvent was then evaporated slowly by heating at 80 °C in a glass vial. Step three—the Bi_2_S_3_ NRs obtained as a black homogeneous mixture was dispersed in 50 mL of DI water and heated it at 80 °C for 30 min while stirring at 500 rpm. Step four −1 mL of a HAuCl_4_·3H_2_O (10 mmol) aqueous solution was added to Bi_2_S_3_-DSPE-PEG_2000_ solution. After 20 min, a reducing agent, 600 µL of ice-cold NaBH_4_ solution in DI water (1 mg/mL) was introduced to initiate the surface-confined decoration of uniform AuNPs while stirring for an additional 30 min at 95 °C at 500 rpm. During this time, the colloidal solution changed in color from black to brownish-black, thereby indicating the formation of AuNPs decorating Bi_2_S_3_ NRs (Au@Bi_2_S_3_NRs). Step five—Au@Bi_2_S_3_ NRs were further purified by centrifugation at 7,000 rpm for 30 min, washed with DI water and dispersed in DI water for further characterization. The resultant AuNPs were typically ~3 nm in diameter, a property that can be easily tuned by modulating the amount of gold ions used. For cell and animal testing, lipid-coated Au@Bi_2_S_3_ NRs were prepared using a thin-film dispersion method. Briefly, DSPE-PEG_2000_ and PC (50:50% w/w) were dissolved in 5 mL of chloroform in a round-bottom flask to afford a thin film upon solvent evaporation, which was subsequently vacuum dried. Au@Bi_2_S_3_ NRs (~ 20 mg), dispersed in cyclohexane and mixed with 1% (v/v) Tween-80, were sonicated. Cyclohexane was evaporated from the Au@Bi_2_S_3_ NRs/cyclohexane/Tween-80 emulsion. A Tween-80-coated Au@Bi_2_S_3_ NRs solution was next added to the lipid film flask and dispersed in a lipid film at 45 °C with bath sonication. The product was collected after centrifugation at 500 rpm for 10 min and then dispersed in MilliQ water for further characterization and CT imaging testing. FITC-DSPE-PEG_2000_ sodium salt was used as a fluorescence conjugate. The FITC-DSPE-PEG_2000_ mass ratios were selected as 0.25% w/w with PC and DSPE-PEG_2000_ to form a lipid film for FITC-Au@Bi_2_S_3_ NRs and to use for cell study. The remaining purification steps of the procedure were the same as above.

### Characterization of Bi_2_S_3_ NRs and Au@Bi_2_S_3_ NRs

The morphology, lattice crystal structure, elemental composition, and chemical color mapping of the synthesized Bi_2_S_3_ NRs and Au@Bi_2_S_3_ NRs were determined by brightfield high-resolution transmission electron microscopy (HR-TEM), selected area electron diffraction (SAED), energy-dispersive X-ray (EDX) spectroscopy, and scanning transmission electron microscopy (STEM) with high-angle annular dark-field (HAADF) (FEI Tecnai Osiris S/TEM). The TEM samples were prepared in cyclohexane and dried onto a copper grid (Electron Microscopy Sciences-EMS, Hatfield, PA, USA) at room temperature. The additional gain in speed can also be used to collect EDX elemental mappings from a larger field-of-view. The lattice fringes of the obtained samples and the corresponding SAED patterns were examined using HR-TEM at 200 kV. The experimental SAED patterns were analyzed using PCED2.0 software.

The particles were also characterized by performing wavelength dispersive X-ray fluorescence (WDXRF) analysis using a Rigaku WDXRF (Supermini200) spectrometer of high-resolution and low detection limits that enabled their elemental analysis. A 200 W, air-cooled, Pd X-ray source was operated at 50 kV and 4 mA to produce excitation spectra with fast elemental detection capability. The system was equipped with a three-crystal analyzing unit to support the standard LiF (200). Nanoparticle surface chemistry analyses was performed with X-ray photoelectron spectroscopy (XPS) and measurements were carried out using monochromatic Al K-alpha (α) X-ray with an energy of 1486.6 eV by Thermo Scientific K-α + XPS (Thermo Fisher Scientific, Waltham, MA, USA). Powder X-ray diffraction (XRD) analyses of Bi_2_S_3_ NRs and Au@Bi_2_S_3_ NRs were performed in the 2θ range of 2–60° using a PANalytical Empyrean diffractometer (PANalytical Inc.; Westborough, MA, USA) with Cu-Kα radiation (1.5418 Å) at 40 keV, 45 mA settings. A mask of 20 mm and a divergence slit of 1/32° were used for the incident beam path. A thin layer of the nanoparticle powder sample was placed on a zero-background silicon plate and the sample holder, which was continuously spun at the rate of 22.5 deg/s during all measurements. The PIXcel3D detector, equipped with a beam monochromator (PANalytical Inc.; Westborough, MA, USA) was scanned at a rate of 0.053 deg/s.

Particle sizes of the Bi_2_S_3_ NRs and Au@Bi_2_S_3_ NRs were determined by measuring hydrodynamic diameter and particle size distribution in water using a Malvern Zetasizer Nano ZS90 (Malvern Panalytical Inc., MA, USA). A Cary 60 UV-Vis Spectrophotometer (Agilent Technologies, CA, USA) was used to characterize the absorbance of the particles.

Excitation and emission spectra of Bi_2_S_3_ NRs and Au@Bi_2_S_3_ NRs were determined using FluoroMax-4 Spectrofluorometer, HORIBA Jobin Yvon. Gold, bismuth, and sulfur quantifications were performed using ICP-MS at the University of Nebraska-Lincoln’s Spectroscopy and Biophysics Core Facility, using an Agilent 7500cx ICP-MS (Santa Clara, CA, USA) coupled with a 96-well plate autosampler Model SC/DX4 from Elemental Scientific, Inc., operating in Mix-Gas collision/reaction mode (3.5 mL H_2_ and 1.5 mL He per minute). Other conditions were plasma power, 1500 W; carrier gas flow, 1 L/minute; makeup gas flow, 0.15 L/minute; sample depth, 8 mm; plasma gas, 15 L/minute. The concentrations were calculated against an external calibration curve with 50 µg/L of Ga, wasused as the internal standard (IS) throughout (Gallium-71 isotope). Tissue samples (liver, spleen, lung, kidney, intestine and pancreas) were suspended in 4 times the volume of analytical grade nitric acid, incubated at room temperature for up to 2 h, followed by overnight digestion at 65 °C. The samples were cooled and diluted 20-fold into the autosampler to reach a 10 mg/mL final concentration.

### Cell toxicity and uptake test

UC-MSCs were cultured in MesenCult™ media supplemented with 2.5% v/v human platelet lysate and 1% v/v penicillin-streptomycin (P/S). The CellTiter-Glo™ assay was used to assess the cytotoxicity of the particles. Briefly, UC-MSCs were seeded at 10,000 cells per well in a 96-well clear bottom plate in the culture medium detailed above. Upon reaching 90% confluence, the cells were washed with PBS, serum-starved in MesenCult™ media without platelet lysate for 4 h, and subsequently replaced with fresh MesenCult™ media containing different concentrations of lipid-coated Au@Bi_2_S_3_ NRs (0.5–200 µg/mL). After incubation for 12 h, the CellTiter-Glo™ assay was performed as per the manufacturer’s instructions. Untreated cells were used as controls. Luminescence was measured using an IVIS Lumina II (Caliper Life Sciences) reader. Data were represented as fold change relative to the control. For determining the cellular uptake of the Au@Bi_2_S_3_ NRs, the UC-MSCs were seeded at 1.5 × 10^6^ cells per well in 12-well clear-bottom plates and cultured to 90% confluency in MesenCult™ media containing 2.5% human platelet lysate and 1% P/S. Subsequently, medium containing FITC-Au@Bi_2_S_3_ NRs at a concentration of 10 µg/mL was added to each well and incubated for 2-12 h. Following incubation, the cells were washed, fixed with 4% v/v paraformaldehyde (PFA), and the nuclei were stained with DAPI. The uptake of fluorescent particles was assessed using Celigo image cytometer (model number 200-BFFL-5 C; Nexcelom Bioscience LLC, CA, USA). To visualize the localization of particles within the cells, confocal microscopy was used. Briefly, cells were seeded on a pre-inserted coverslip in cell culture plate wells. Following the attachment of cells, the particles were added at a concentration of 10 µg/mL. After incubation for 12 h, the cells were fixed in 4% PFA, the nuclei were stained with DAPI, and imaged using a confocal microsocpe (Leica TSC SP8X White Laser Confocal Microscope).

### CT Imaging of particles

To assess Au@Bi_2_S_3_ NRs at relevant CT imaging levels with Bi_2_S_3_ NRs, Au@Bi_2_S_3_ NRs were dispersed in DI water with different mass concentrations ranging from 11.4, 22.8 45.5, 91.2, to 182.3 µg/mL based on the bismuth concentration. ICP-MS analyses of lipid-coated Au@Bi_2_S_3_ NRs suspension were shown with metal components: S = 283.48 µg/mL, Au = 440.11 µg/mL and Bi = 3,647.83 µg/mL concentration. Phantoms were scanned using a small animal scanner, Siemens Inveon PET/CT system (Siemens Medical Solutions, Knoxville, TN USA) with scanning parameters listed in the next section.

### Biodistribution

Rats (8–12 weeks old, male, Wistar) were purchased from Charles River Laboratories (USA) and housed according to Stanford University’s Administrative Panel for Laboratory Animal Care (APLAC). All procedures were performed in accordance with the regulations approved by the Institutional Animal Care and Use Committee (IACUC) of Stanford University. A total of 2 mL of lipid-coated FITC-Au@Bi_2_S_3_ NRs with an ICP-MS quantitative S = 283.48 µg/mL, Au = 440.11 µg/mL, Bi = 3647.83 µg/mL concentration was injected intravenously (IV). Following the injection, the rats were anesthetized with isoflurane (3–4% for induction and 1–3% for maintenance) and imaged with CT after Au@Bi_2_S_3_ NRs administration using the Siemens Inveon PET/CT system (Siemens Medical Solutions, Knoxville TN USA). CT settings were a 121 projection and SB70 mm installed pallet. X-ray source conditions were applied for 79 keV tube voltage, 493 µA of tube current, 50 µm of spot size, 0.5 mm of filter, 18.37 × 12.2 cm of FOV, 2.45 of scale, 482.26-150.17, Hot metal of scale bar. Inveon Acquisitions workplace viewer 4.0 software was used to acquire data. All animals were euthanized 120 h post-injection of the Au@Bi_2_S_3_ NRs, using carbon dioxide as the method of euthanasia.

### Histological analysis

Histological analysis of the explanted organs (liver, spleen, pancreas, lung, and kidneys) at 120 h following intravenous injection of Au@Bi_2_S_3_ NRs was performed to assess the biocompatibility of our particles in small animals. The excised organs were fixed in 10% formalin, embedded in paraffin, sliced, and stained with hematoxylin and eosin (H&E). Furthermore, immunohistochemistry using CD45 staining to assess any inflammatory response to Au@Bi_2_S_3_ NRs was performed, as previously described [33]. The stained sections were analyzed using a NanoZoomer slide scanner 2.0-RS (Hamamatsu Photonics, Japan).

## Results and discussion

### Au@Bi_2_S_3_ NRs synthesis and characterization

Solvothermal synthesis was used to synthesize Bi_2_S_3_ NRs by mixing bismuth and sulfur precursors, such as thioacetamide and bismuth neodecanoate, dissolved in an organic solvent. The resulting solution was then placed in a high-pressure reactor autoclave and heated to the desired reaction temperature. As the bismuth and sulfur precursors react, they form bismuth sulfide composites, which selectively precipitates as Bi_2_S_3_ NRs. The resulting mixture was then cooled, and Bi_2_S_3_ NRs were collected by centrifugation. Next, gold nanoparticles (AuNPs) were decorated in situ onto the surface of Bi_2_S_3_ NRs by reducing HAuCl_4_·3H_2_O with NaBH_4_ to create Au@Bi_2_S_3_ NRs. Here, gold ions, such as Gold (III) chloride trihydrate, were reduced onto the surface of Bi_2_S_3_ NRs using sodium borohydride. Biocompatible hydrophilic NPs were then developed with the help of a uniform lipid coating (DSPE-PEG_2000_ and phosphatidylcholine) using a film hydration method (Schematic 1); this reaction is carried out in an aqueous solution with the final Au@Bi_2_S_3_ NRs purified for characterization using high-resolution transmission electron microscopy (HR-TEM), scanning transmission electron microscopy (STEM), selected area electron diffraction (SAED), X-ray diffraction (XRD), X-ray photoelectron spectroscopy (XPS), X-ray fluorescence (XRF), and inductively coupled plasma mass spectrometry (ICP-MS).

**Schematic 1:**
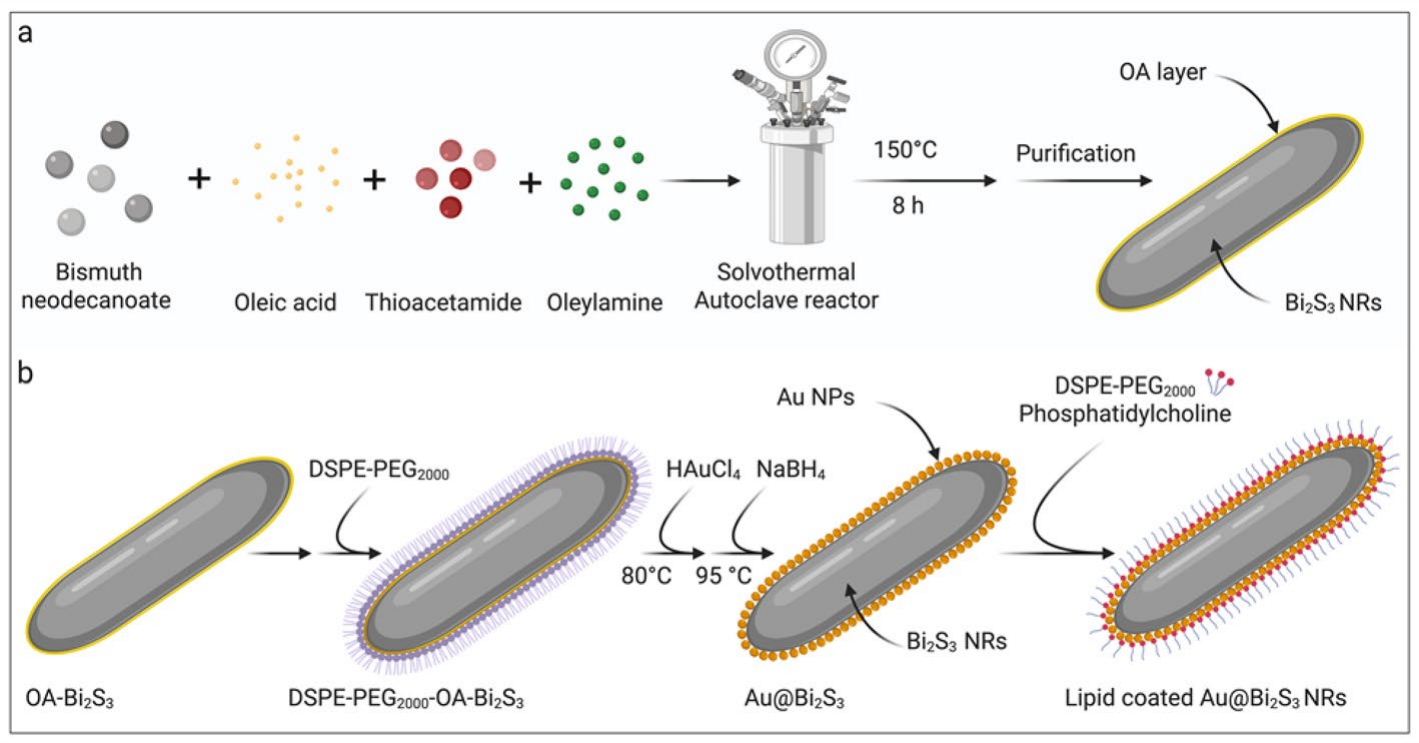
Schematic for the synthesis of Au@Bi_2_S_3_ NRs. Multimodal Au@Bi_2_S_3_ NRs for high contrast X-ray CT imaging were obtained by core-shell structural synthesis. **a** Bi_2_S_3_ NR cores were synthesized using a solvothermal synthetic route. **b** Bi_2_S_3_ NRs were stabilized with a lipid coating, consisting of phosphatidylcholine and DSPE-PEG_2000_ before being coated with AuNPs on its surface to create Au@Bi_2_S_3_ NRs.

#### HR-TEM, SAED and STEM

**Fig. 1:**
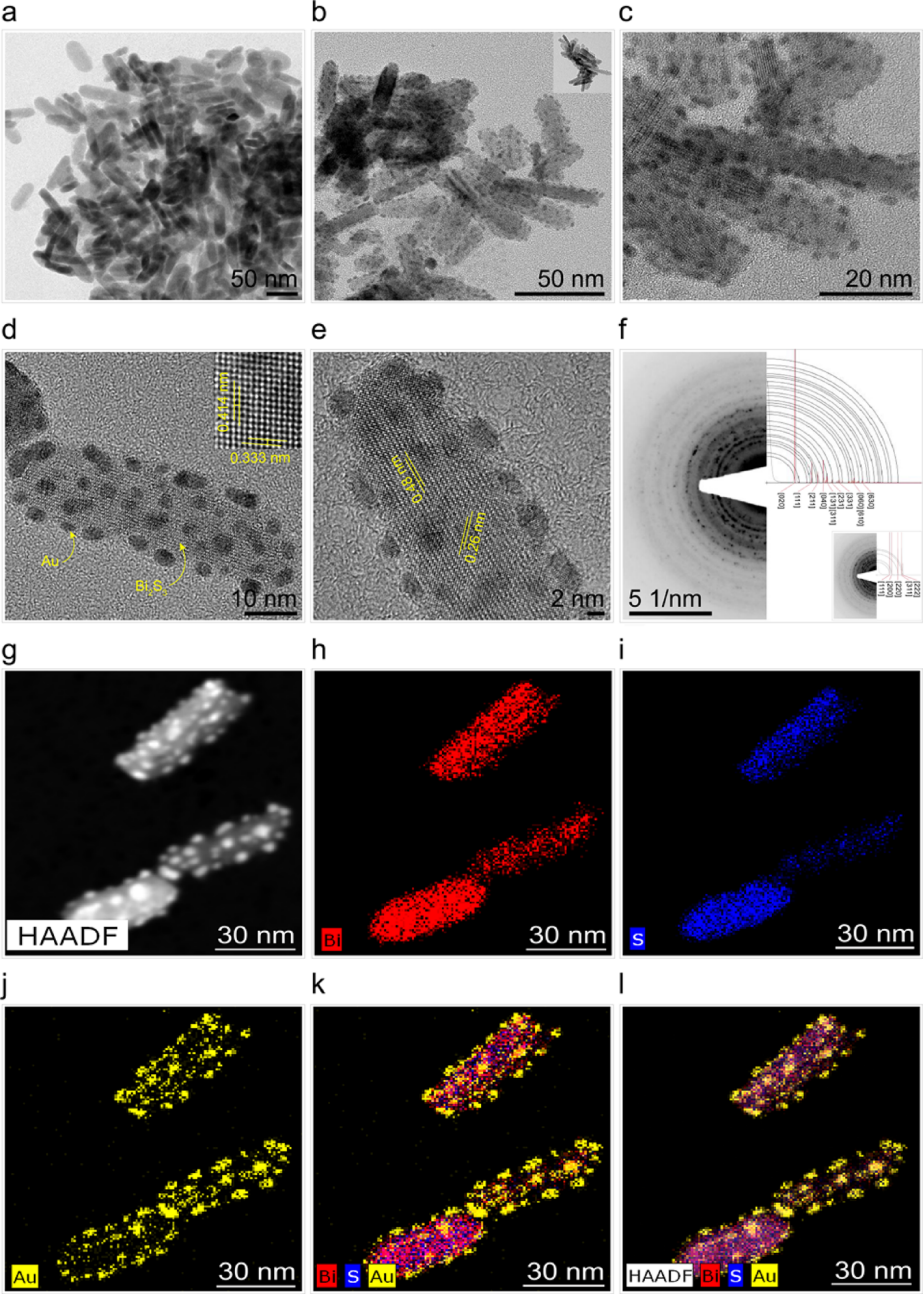
Characterization of Au@Bi_2_S_3_ NRs. Low-resolution TEM images show **a** Bi_2_S_3_ NRs and **b** AuNP-decorating Bi_2_S_3_ NRs (Au@Bi_2_S_3_ NRs). **c** Magnified TEM image displaying AuNPs distributed uniformly across the Bi_2_S_3_ NR surface. **d**–**e** HR-TEM images of Au@Bi_2_S_3_ NRs showing surface Au and the Bi_2_S_3_ core; the inset displays lattice fringes with measured spacings of 0.414 nm, 0.333 nm, 0.260 nm, and 0.480 nm, corresponding to Bi_2_S_3_ crystallographic planes (220, 130, 311, and 210). **f** Selected-area electron diffraction (SAED) pattern of Au@Bi_2_S_3_ NRs with indexed diffraction rings consistent with expected interplanar spacings. **g** HAADF-STEM image of Au@Bi_2_S_3_ NRs. Elemental STEM maps show the distribution of **h** Bi, **i** S, and **j** Au. **k**–**l** Overlay maps combining Bi, S, and Au elements alongside the corresponding HAADF signal

HR-TEM was used to examine the crystal structure and lattice defects of Au@Bi_2_S_3_ NRs, as well as their size, shape, and distribution. Low-power TEM images demonstrated that Bi_2_S_3_ NRs and Au@Bi_2_S_3_ NRs were of a uniform population having a rod shape of ~ 10 nm width and ~ 33–43 nm length (*N* = 20) (Fig. 1a–c). HR-TEM images showed AuNPs with a uniform spherical size (~ 3–5 nm) that were homogeneously distributed and attached to the surface of Bi_2_S_3_ NRs (Fig. 1d–e). Furthermore, HR-TEM data also showed a lattice pattern with atoms at interplanar distances of 0.414 nm, 0.480 nm, 0.333 nm, and 0.260 nm, corresponding to the (220), (210), (130), and (311) planes, respectively (JCPDS 01-089-8963), which are in accordance with the atomic configuration of the Au@Bi_2_S_3_ NRs. The SAED patterns of Au@Bi_2_S_3_ NRs are shown by simulation indexation. The simulation index lines correspond to the rod’s interplanar spacing (Fig. 1f) [4]. SAED confirmed that the AuNPs were coordinated with the bismuth template on the surface of the Bi_2_S_3_ NRs, which was assigned to the (111), (200), (220), (311), and (222) plane of gold (JCPDS; 04-007-4652; 03-065-3093 and 01-071-4073) for a face-centered cubic gold structure. The simulation indexation of the orthorhombic Bi_2_S_3_ SAED pattern shows planes of (020), (211), (040), (131), (311), (231), (331), (060), (610), and (630) (JCPDS; 01-089-8963; 04-019-2866) [4, 30], all of which confirm that Au@Bi_2_S_3_ NRs were efficiently synthesized. High-angle annular dark-field scanning transmission electron microscopy (HAADF-STEM) illustrated the particle heterostructure and distribution of the elements of bismuth (red), sulfur (blue), and gold (yellow) with their mapping showing that the core of the particles was composed of Bi and S, while the shell was composed of Au (Fig. 1g–l).

#### Structural characterization: EDX and XRD

**Fig. 2:**
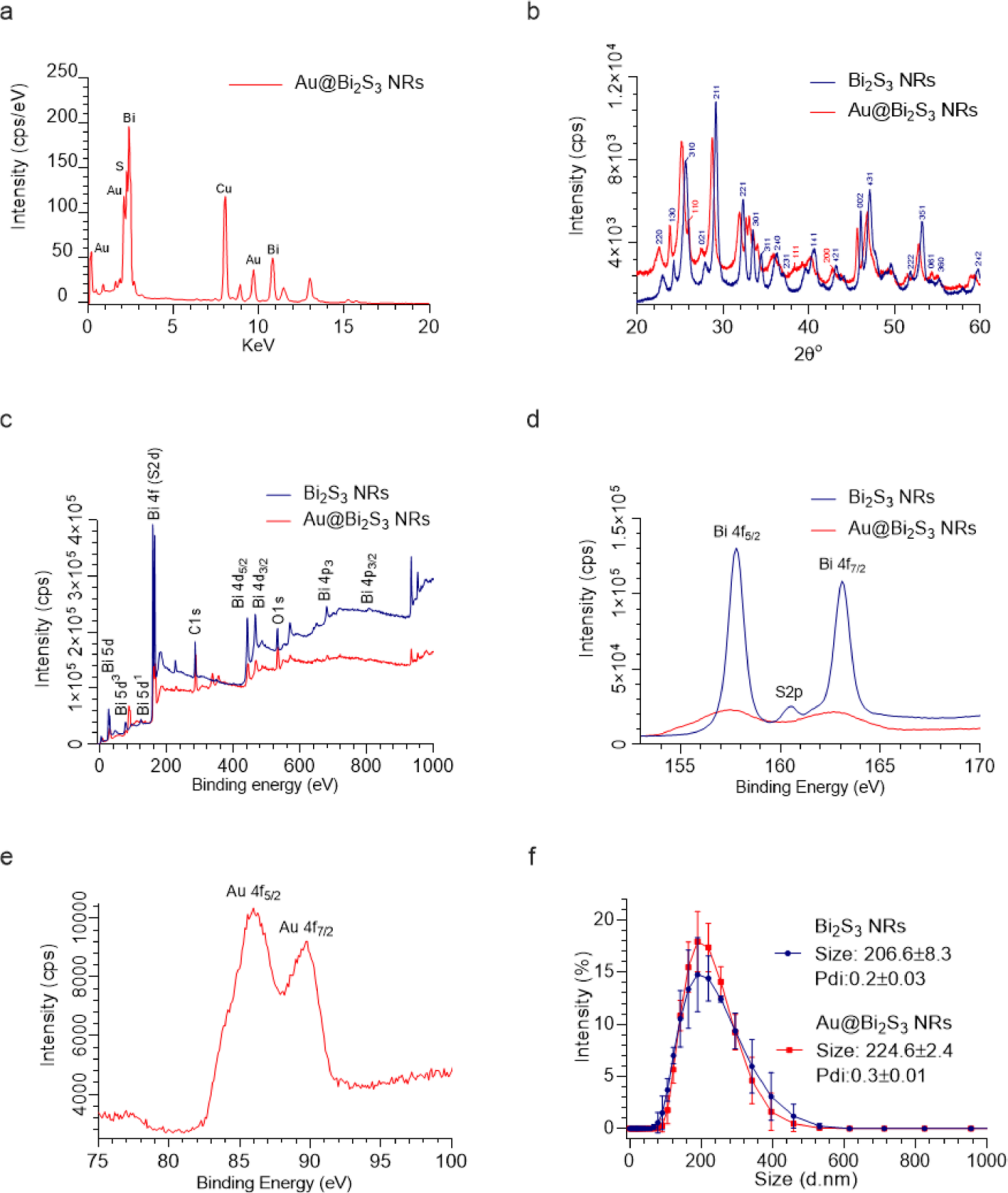
Structural and surface chemistry characterization of particles. **a** Energy-dispersive X-ray (EDX) elemental mapping of Bi, S, and Au, with Cu originating from the TEM grid. **b** X-ray diffraction (XRD) patterns of Bi_2_S_3_ NRs and Au@Bi_2_S_3_ NRs with indexed peaks corresponding to expected crystallographic planes. **c**–**e** X-ray photoelectron spectroscopy (XPS) analysis: **c** full survey spectrum, **d** Bi region, and **e** Au region of Au@Bi_2_S_3_ NRs. **f** Hydrodynamic size distribution of Bi_2_S_3_ NRs and Au@Bi_2_S_3_ NRs measured by dynamic light scattering (DLS)

Energy dispersive X-ray spectroscopy (EDX) can identify the elemental composition of NPs by measuring the energy and intensity of X-rays emitted from the sample when it is bombarded with high-energy electrons; this technique provides detailed information about the structure and elemental composition of nanomaterials. EDX spectroscopy analysis demonstrated Bi, S, and Au signals with signature energy peaks in Au@Bi_2_S_3_ NRs (Fig. 2a). It is important to note that EDX spectroscopy is a surface analysis technique that only provides information about the top few atomic layers of Au@Bi_2_S_3_ NRs. As such, we also employed XRD to obtain additional detailed information about the crystal structure of Au@Bi_2_S_3_ NRs. Specifically, the XRD diffraction pattern was used to both identify the presence of specific phases in the Au@Bi_2_S_3_ NRs and to determine the crystal structure of those phases. The crystal structure and lattice planes of Bi_2_S_3_ NRs and Au@Bi_2_S_3_ NRs were assessed using powder XRD studies. The XRD pattern of plain (undecorated) Bi_2_S_3_ NRs showed signature peaks at 23.16° (220), 24.29° (130), 25.71° (310), 28.11° (021), 29.19° (211), 32.39° (221), 33.50° (301), 34.50° (311), 36.40° (240), 38.63° (231), 40.77° (141), 43.30° (421), 46.10° (002), 47.16° (431), 53.29° (222), 54.9° (351), 55.3° (061), 55.42° (360), and 59.23° (242), corresponding to a d-spacing of 0.396 nm, 0.356 nm, 0.352 nm, 0.324 nm, 0.310 nm, 0.280 nm, 0.271 nm, 0.263 nm, 0.251 nm, 0.245 nm, 0.225 nm, 0.211 nm, 0.198 nm, 0.194 nm, 0.177 nm, 0.173 nm, 0.167 nm and 0.155 nm, respectively, all which accompany an orthorhombic structure (JCPDS: 01-089-8963) for the Bi_2_S_3_ NRs. For Au@Bi_2_S_3_ NRs, along with the Bi_2_S_3_ NRs peaks as mentioned above, additional peaks at 25.26° (110), 36.40° (111) and 43.30° (200), were observed for the planes that correspond to 0.355 nm, 0.235 nm, and 0.203 nm, respectively (JCPDS: 04-007-4652; 01-071-4073). XRD patterns with no anomalous peaks indicated that the synthesized nanomaterials were free from contaminants (Fig. 2b). It is important to note that XRD is a non-destructive technique, and as such, is not capable of providing information about the chemical composition of Au@Bi_2_S_3_ NRs. To determine the chemical composition of Au@Bi_2_S_3_ NRs, we used other techniques such as XPS, XRF spectrometer, and ICP-MS.

#### Chemical characterization: XPS, XRF, and ICP-MS

XPS is a surface-sensitive analytical technique that measures the elemental composition and chemical states of the elements present at the surface of Au@Bi_2_S_3_ NRs (Fig. 2c–e). The spectrum survey revealed the presence of elements, such as Bi, S, O, and C, indicating the high purity of the particles. The peak for O arises, presumably, from adsorbed gases and/or oxides of C on the surfaces of the samples [34]; this observation is common in the case of ultrafine powder samples when exposed to atmospheric conditions. From our XPS analyses of Au@Bi_2_S_3_ NRs, we found that the main peaks we observed are due to the Bi, S, and S2p orbitals and Bi 4f peaks. High-resolution XPS spectra of Au 4f and Bi 4f (Fig. 2d and e) further elucidated the composition of the Au@Bi_2_S_3_ NRs. For each asymmetric Bi 4f7/2 or Bi 4f5/2, the peak in both Bi_2_S_3_ and Au@Bi_2_S_3_ NRs could be deconvoluted into two peaks in the high-resolution XPS spectra in the Bi region (Fig. 2d). Peaks at 157.78 and 163.08 eV, which can be assigned to the binding energies of Bi 4f5/2 and Bi 4f7/2 in Bi_2_S_3_ NRs, respectively, belong to lattice Bi bound to S (Bi-S bond). However, in Au@Bi_2_S_3_ NRs, both peaks had shifted toward a lower intensity, meaning some Au atoms in Au@Bi_2_S_3_ NRs may bind to S atoms to form an Au-S bond, which is in line with thiols demonstrating a high affinity for Au. The high-resolution XPS spectra in the Au region (Fig. 2e) showed two peaks at 85.88 and 89.68 eV, which can be assigned to the binding energies of Au 4f5/2 and Au 4f7/2 in Au@Bi_2_S_3_, respectively, thereby indicating the presence of Au in the Au@Bi_2_S_3_ NRs nanostructures. The broad peak at around 224.68 eV corresponds to the binding energy of S2s (Fig. S1a). Figure S1b shows the peaks of S 2p3/2 and S 2p1/2 located at 165.58 and 160.28 eV, respectively, which match the literature values of the sulfide anion (S^2−^) (Fig. S1b). The observed values were found to be in close agreement with data reported by Grigas et al. [35]. Collectively, the data showed that Au@Bi_2_S_3_ NRs have a uniform composition with bimetallic Bi/S cores and Au shells. Overall, XPS is a powerful tool for studying the surface chemistry of Au@Bi_2_S_3_ NRs and provides valuable information about the elemental composition of the 2-3 outermost layers comprising the Bi_2_S_3_ NRs and that of Au@Bi_2_S_3_ NRs. In addition to their chemical makeup and structural arrangements, we implemented the technique of XRF to evaluate the elemental mass distribution of Au, Bi, and S. As anticipated, both the Bi_2_S_3_ and Au@Bi_2_S_3_ NRs exhibited Bi/S = 83.49/15.84 mass percent (%) , and Bi/S/Au = 74.53/16.69/8.77 mass percent (%) (Au for only the Au@Bi_2_S_3_ NRs, respectively (Fig. S2). Moreover, Bi/Au ratio = 8.28:1% w/w content in lipid-decorated Au@Bi_2_S_3_ NRs was determined by ICP-M S. As the Bi/Au ratio obtained from performing ICP-MS squarely aligns with that found using XPS and XRF, such earlier data bolsters and explicily confirms the elemental ratios of both the Bi_2_S_3_ and Au@Bi_2_S_3_ NRs.

#### Particle size and zeta potential analysis

Dynamic light scattering (DLS) was used to evaluate the average size, polydispersity, and zeta potential of particles (Fig. 2f). The particle size of Au@Bi_2_S_3_ NRs and Bi_2_S_3_ NRs were 224.6 ± 2.4 nm and 206.6 ± 8.3 nm, respectively. The particle size of Au@Bi_2_S_3_ NRs was ~ 20 nm bigger than the size of Bi_2_S_3_ NRs because of the AuNPs decoration. The polydispersity index, which measures the sample’s heterogeneity based on size, was comparable between the particles (0.2 and 0.3 for Bi_2_S_3_ NRs and Au@Bi_2_S_3_ NRs, respectively). Moreover, the DLS showed an average zeta potential of -26.46 ± 2.43 mV for the Bi_2_S_3_ NRs and − 45.76 ± 1.02 mV for Au@Bi_2_S_3_ NRs, which demonstrates an increase of ~-20 mV for Au@Bi_2_S_3_ NRs when compared to that of the Bi_2_S_3_ NRs.

The higher negative surface charge of Au@Bi_2_S_3_ NRs relative to undecorated Bi_2_S_3_NRs is attributed to the DSPE-PEG_2000_ phospholipid coating introduced during the gold nanoparticle seeding step. The phosphate headgroups of the lipid contribute to electrostatic charge, while the PEG chains extend the hydrodynamic shear plane, which provides additional steric stabilization. These combined electrostatic and steric effects contribute to enhanced colloidal stability in aqueous environments. Formal colloidal stability testing of Au@Bi_2_S_3_ NRs in PBS and serum-containing media over extended time points was not performed in this proof-of-concept study. However, gold nanoparticles are widely regarded as chemically stable and resistant to aggregation under physiological conditions, and DSPE-PEG_2000_ coatings are well established to confer colloidal stability through combined steric and electrostatic effects. Based on these established properties, we expect the lipid-PEGylated Au@Bi_2_S_3_ NRs to exhibit stability consistent with literature [36, 37]. Systematic evaluation in biological media will be performed in future studies.

DLS reports an intensity-weighted hydrodynamic diameter that is highly sensitive to small populations of clusters. The observed Z-average is substantially larger than the rod dimensions observed by TEM, indicating that the particles likely form nanoscale clusters in aqueous suspension despite appearing individually dispersed in dried TEM grids. The DSPE-PEG_2000_ and phosphatidylcholine lipid coating as well as the anisotropic shape contribute to an increased apparent size, but the magnitude of the difference suggests that clustering/association dominates the DLS signal. Also, DLS reports an intensity-weighted size distribution, which disproportionately emphasizes larger species in solution, further contributing to the observed increase in apparent particle size. These factors collectively explain the difference between TEM and DLS measurements and are consistent with behavior reported for other PEGylated nanomaterials [38–41].

### Cellular uptake and toxicity evaluation

To evaluate the biocompatibility of Au@Bi_2_S_3_ NRs, we tested their cellular uptake and toxicity using Umbilical Cord (UC) - Wharton's Jelly derived mesenchymal stem cells (MSCs), given the potential to use these nanoparticles to label and track these cells. Luminescence-based CellTiter-Glo™ assays were used to test UC-MSC viability after exposure for 12 h to different concentrations of Au@Bi_2_S_3_ NRs (0.5 to 200 µg/mL). Viable cells produced luminescent signal while reduced luminescence indicated decreased viability. Cell viability remained about 100% at 0.5-40 µg/mL, but decreased markedly at higher concentrations (60–200 µg/mL) (Fig. S3a,b). Next, cellular uptake of FITC-Au@Bi_2_S_3_ NRs was studied. Representative confocal images of FITC-Au@Bi_2_S_3_ NRs (10 µg/mL) incubated with UC-MSCs for 12 h are reported in Fig. S3c, which shows green fluorescence in the cell cytoplasm (FITC staining of the particles) and blue nuclei (DAPI staining of cell nuclei). To further verify in vitro uptake of Au@Bi_2_S_3_ NRs in UC-MSCs, FITC-Au@Bi_2_S_3_ NRs cellular uptake and intracellular localization of particles within UC-MSCs were assessed using a Celigo Imaging Cytometer in a time-series dynamic study (up to 12 h). Cellular uptake of FITC-Au@Bi_2_S_3_ NRs was observed to occur in a time-dependent manner. Images show an increased amount of particle aggregates inside the cytoplasm of UC-MSCs over time (after 2 h through 12 h), as noted by the increased number of black particles or yellow dots in the bright-field and fluorescence images, respectively (Fig. S3d). Particle uptake was relatively slow during the first few hours of incubation before reaching saturation at 10–12 h (Fig. S3d). It is important to note that the uptake of Au@Bi_2_S_3_ NRs by UC-MSCs, as well as their potential effects on UC-MSCs, can vary depending on the specific study and the conditions used. Previous studies show that various factors such as the size, surface properties, concentration, and exposure time affect the particle uptake in cells and tissue [4], and their cellular uptake can occur through several mechanisms, including endocytosis, phagocytosis, and pinocytosis [1, 4, 42].

### In vivo biodistribution and clearance

**Fig. 3:**
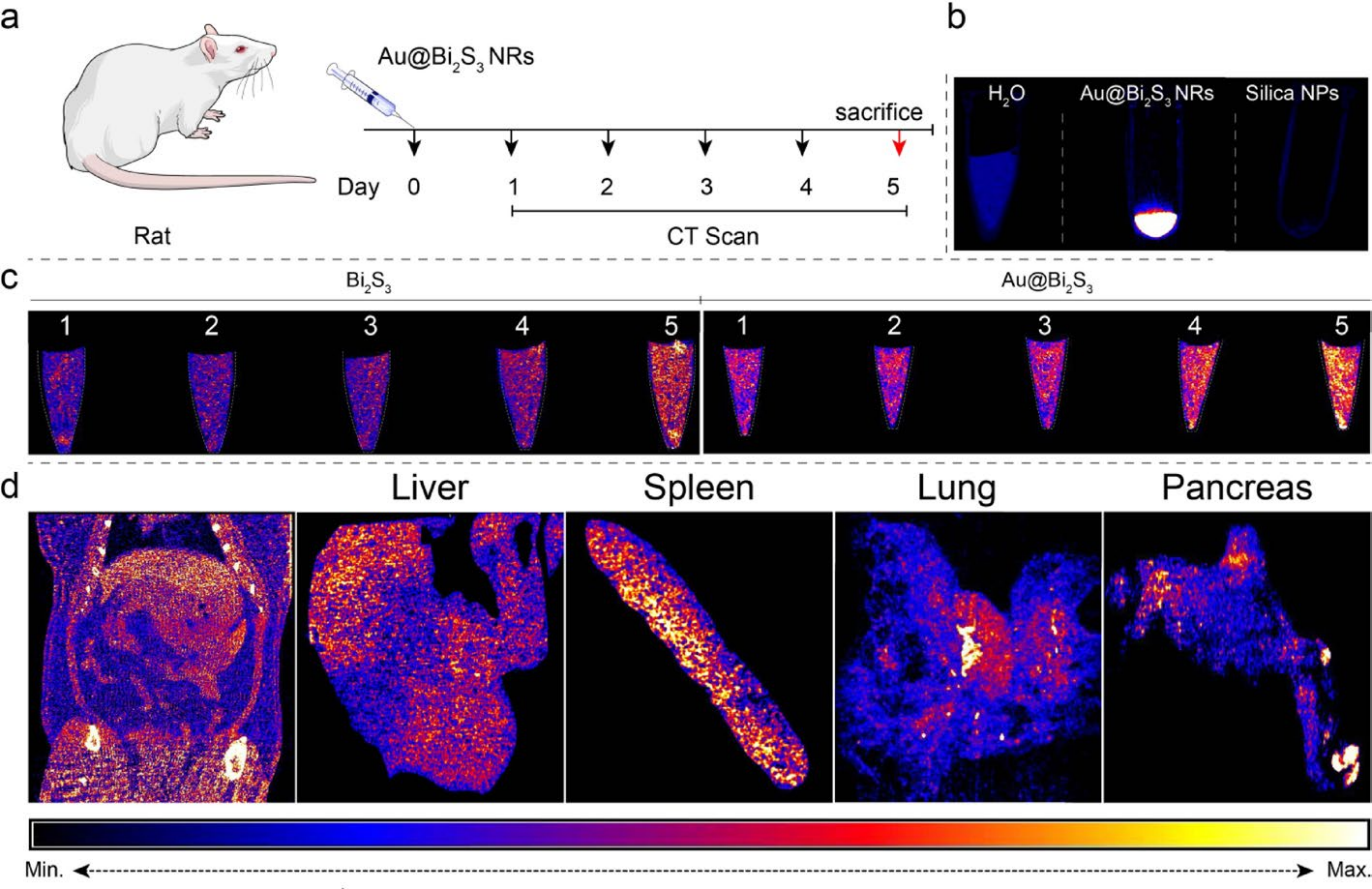
In vivo biodistribution of Au@Bi_2_S_3_ NRs. **a** Experimental timeline for CT imaging studies in Wistar rats following intravenous administration of Au@Bi_2_S_3_ NRs. **b** CT images of Au@Bi_2_S_3_ NRs compared with silica nanoparticles (SiNPs) powder and water controls. **c** CT phantom images of Bi_2_S_3_ NRs and Au@Bi_2_S_3_ NRs prepared at concentrations of 11.4, 22.8, 45.6, 91.2, and 182.3 µg/mL (left-to-right) in PBS. **d** Coronal-plane in vivo CT images acquired 120 h after IV injection of Au@Bi_2_S_3_ NRs. Corresponding whole body and ex vivo CT images of harvested liver, spleen, lung, and pancreas collected at 120 h. CT image display scale: minimum 150 HU to maximum + 482 HU (Hot Metal color scale; panel **b**, **c** and **d**).

The feasibility of utilizing Au@Bi_2_S_3_ NRs as a contrast agent for visualizing soft tissues was assessed through CT imaging (Fig. 3a). Initially, we compared the contrast efficacy of Au@Bi_2_S_3_ NRs with that of mesoporous silica nanoparticles (SiNPs) in dry powder form (Fig. 3b). Our results demonstrated that the combination of bismuth and gold in Au@Bi_2_S_3_ NRs produced significantly higher contrast levels compared to both Bi_2_S_3_ NRs and SiNPs. (Fig. 3b-c).

Next, we investigated the in vivo biodistribution of Au@Bi_2_S_3_ NRs following intravenous injection in rats, utilizing whole-body CT imaging with static scans conducted at various time points: 24, 48, 72, 96, and 120 h post-injection. The data presented here focuses exclusively on the 120 hour time point, excluding information from the other time intervals. The 120-hour post-injection scan (Fig. 3d) revealed that Au@Bi_2_S_3_ NRs predominantly localized in major organs, including the liver, spleen, lung, and pancreas. This finding was further validated through ex vivo imaging of the harvested organs (Fig. 3d), which were collected at 120 h after particle injection. Notably, the signal intensity at 120 h in the liver and spleen was significantly stronger compared to that in the lung and pancreas, a trend corroborated by ICP-MS measurements. Additionally, ICP-MS results indicated that the bismuth content in various organs aligned with the signal intensity observed in CT imaging at the 120-hour mark. Accumulation of Au@Bi_2_S_3_ NRs was confirmed in organs, with bismuth concentrations measured at 893.3 ± 304.5 µg/g in the spleen, 714.5 ± 177.3 µg/g in the liver, 126.4 ± 50.6 µg/g in the lung, 74.9 ± 5.3 µg/g in the kidney, 4.0 ± 1.5 µg/g in the intestine, 7.8 ± 3.0 µg/g in the pancreas. (Fig. S4).

 Hence, the extended imaging window following intravenous administration of Au@Bi_2_S_3_ NRs presents significant potential for various clinical applications.

The CT imaging data demonstrate a clear concentration-dependent increase in X-ray attenuation for Au@Bi_2_S_3_ nanorods across the tested range of 11.4 to 182.3 µg/mL. Visual inspection of the CT phantom images reveals a monotonic increase in signal intensity with increasing nanoparticle concentration, consistent with the expected relationship between material density and X-ray attenuation. The displayed Hounsfield Unit (HU) scale, ranging from 150 to + 482 HU, indicates that the nanoparticles generate substantial contrast enhancement within clinically relevant imaging ranges. This trend is consistent with a near-linear relationship between nanoparticle concentration and attenuation, as predicted by X-ray attenuation theory and widely reported for high atomic number nanomaterials. These findings confirm the strong attenuation capability of Au@Bi_2_S_3_ NRs and support their potential as effective CT contrast agents.

The biodistribution and subsequent biological effects of NPs depend on a variety of factors, including their size, shape, core materials, and method of administration, to name a few. Following IV administration, Au@Bi_2_S_3_ NRs, are predominantly taken up by the liver and spleen, as also shown by several other studies that have examined Bi_2_S_3_ NRs [4, 43, 44]; this is in keeping with the function of organs of the reticuloendothelial system, of which the liver and spleen are part of, which remove and eliminate foreign substances from the bloodstream. Once in these organs, NPs are then processed. Here, there will be a balance between NPs clearance and potential ongoing inflammation (due to how the body processes the particles) that could result in potential local cellular injury [4, 45–47].

### Histological assessments

**Fig. 4:**
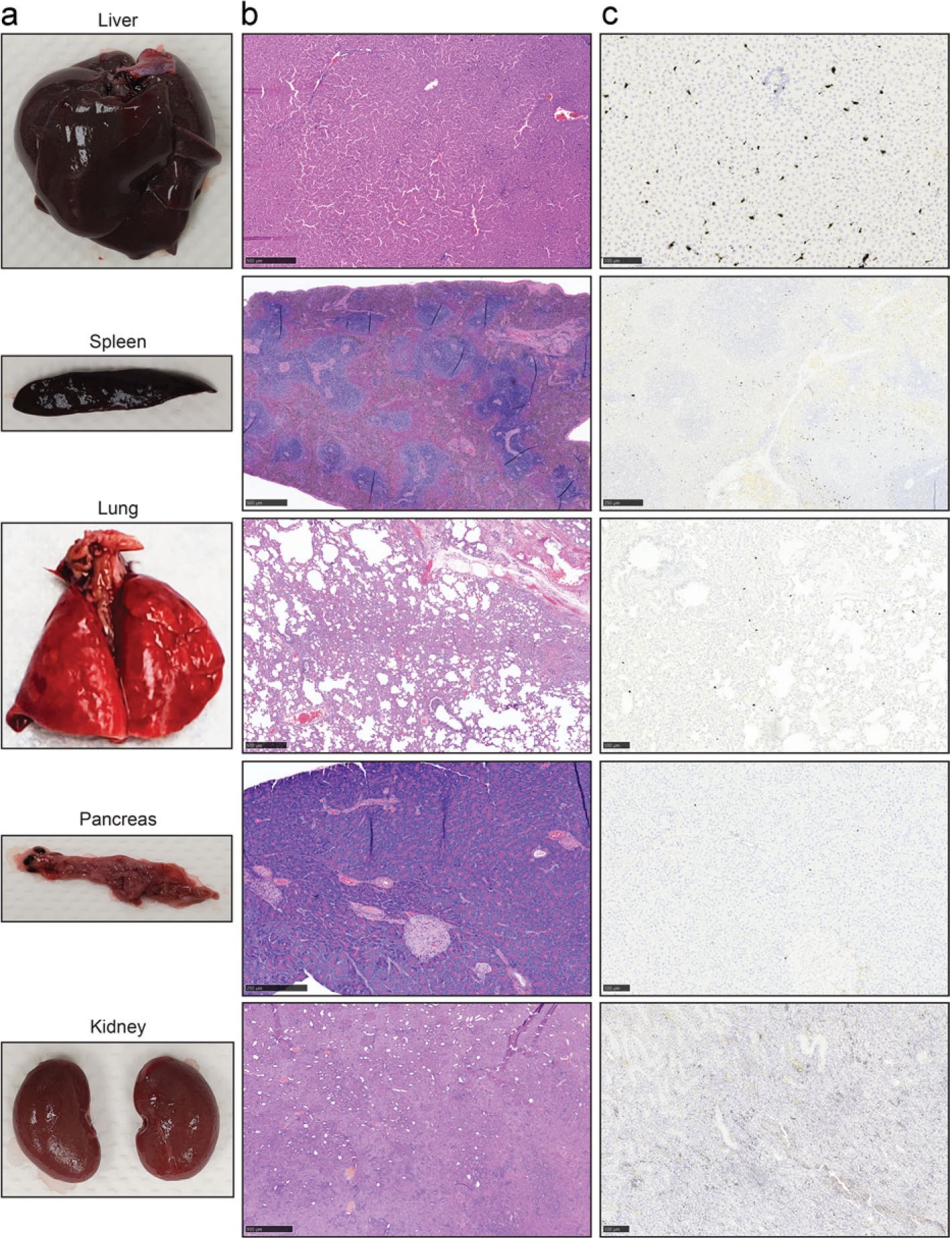
Histological assessment. **a** Representative photograph of excised organs collected 120 h after IV administration of Au@Bi_2_S_3_ NRs. **b** Hematoxylin and eosin (H&E) staining of liver, spleen, pancreas, lung, and kidney sections. **c** Immunohistochemical staining for CD45 using DAB (3,3′-diaminobenzidine) chromogen. Black punctate signal in H&E sections indicate intracellular particle deposits. CD45 staining highlights leukocyte presence within tissue sections

The liver, spleen, lung, kidney, and pancreas demonstrated grossly normal size and morphology at the end of our study. Subjectively, Au@Bi_2_S_3_ NRs increased the darkness of the color of the liver and spleen compared to control animals (Fig. 4a) with H&E-stained samples (Fig. 4b) showing particle deposits within tissue sections, most prominently in liver and spleen, without obvious surrounding inflammatory cell infiltrate at 5 days post-administration. Immunohistochemical staining, using CD45 (Fig. 4c), also indicated no leukocyte infiltration. Collectively, Au@Bi_2_S_3_ NRs elicited minimal-to-no inflammatory response in the short term. Future studies will aim to evaluate how these organs will handle Au@Bi_2_S_3_ NRs to determine their long-term safety profile.

## Conclusion

In this work, we developed a simple solvothermal strategy to synthesize Au@Bi_2_S_3_ nanorods (NRs) and performed comprehensive, state-of-the-art physicochemical characterization of the resulting hybrid nanostructures. By using the lipid-based phase-changing agent DSPE-PEG_2000_ as a templating/functionalization approach to decorate Bi_2_S_3_ NRs with AuNPs—rather than Tween 20 as previously reported [39], we achieved improved control over particle size, crystallinity, and batch-to-batch reproducibility. Importantly, Au@Bi_2_S_3_ NRs enhanced soft-tissue contrast in CT imaging in living animals, supporting their promise as a platform for further development toward multimodal imaging and theranostic applications.

Although this study did not include a direct experimental comparison with conventional iodinated contrast agents, it is well established that high–atomic number materials such as gold and bismuth provide substantially higher mass-normalized X-ray attenuation than iodine due to stronger photoelectric absorption [48, 49]. Moreover, iodinated agents are rapidly cleared, limiting imaging windows and often necessitating precise bolus timing or repeated dosing. In contrast, the Au@Bi_2_S_3_ NRs described here demonstrated sustained in vivo retention and prolonged imaging capability, which may offer practical advantages for specific diagnostic workflows. Future studies will focus on direct, quantitative benchmarking against clinically used iodinated agents (e.g., iohexol), alongside expanded safety, pharmacokinetic, and application-specific evaluations to further contextualize performance and accelerate translation.

## Supplementary Information

Below is the link to the electronic supplementary material.

Supplementary Material

## Data Availability

The authors confirm that the data supporting the findings of this study are available within this article and its Supplementary material. Raw data that support the findings of this study are available from the corresponding author upon reasonable request.
