# Supplementary material for: Gold decorated bismuth sulfide nanorods for enhanced computed tomography imaging with in vitro and in vivo validation
Source: Discov Nano. 2026 Jun 10;21(1):253. doi: 10.1186/s11671-026-04708-1 (PMC13253923; doi:10.1186/s11671-026-04708-1)
Supplement: Supplementary file 1 — Supplementary Material [file 11671_2026_4708_MOESM1_ESM.docx]

**SUPPLEMENTARY FIGURE**

**Figure S1. X-ray Photoelectron Spectroscopy (XPS) analysis of Au@Bi_2_S_3_ NRs.** S2s region **(a)** and S2p regions **(b)** of Au@Bi_2_S_3_ NRs, respectively, are depicted.

**Figure S2.XRF analysis of Bi_2_S_3_ and Au@Bi_2_S_3_ NRs**

**Figure S3. *In vitro* evaluation of Au@Bi_2_S_3_ NRs in UC-MSCs: viability and cellular uptake.** **(a)** Representative CellTiter-Glo^TM^ luminescence plate images of UC-MSCs after 12h treatment with Au@Bi_2_S_3_ NRs (0-200 µg/mL). Pseudocolor indicates luminescence intensity (radiance). **(b)** Plot showing quantitative luminescence radiance from UC-MSCs treated with Au@Bi_2_S_3_ NRs and normalized to untreated UC-MSCs (control). Data represent mean ± SD; n=6 independent replicates. Error bars indicate standard deviation. Cell viability expressed as fold change relative to untreated control. Assay: CellTiter-Glo™ luminescence. **(c)** Figure showing representative confocal images of UC-MSCs after treatment with FITC-Au@Bi_2_S_3_ NRs (10 µg/mL). The confocal images show cell nuclei in blue (DAPI staining), Fluorescein-5-isothiocyanate (FITC) labeled-Au@Bi_2_S_3_ NRs exhibits in green fluorescence and the merge indicates the combination of green and blue. **(d)** Time-dependent uptake of FITC-Au@Bi_2_S_3_ particles in UC-MSCs at different time points (2-12h) after treatment with FITC-Au@Bi_2_S_3_ NRs (10 µg/mL). The images were acquired using a Celigo equipment. Representative images show cell nuclei in blue (DAPI staining), cells as bright-field, particles in green and the merge indicates the combination of green and blue fluorescence.

**Figure S4. ICP-M analysis of the concentration of bismuth in excised tissues.** A single IV injection of Au@Bi_2_S_3_ NRs was given to Wistar rats. Organs were collected at 120 h. Bismuth uptake is reported as µg Bi per g tissue. Data are shown as mean ± SEM (n = 4 per organ, except intestine n = 3). Statistical analysis was performed using one-way ANOVA followed by Tukey’s post hoc test. Differences were considered significant at p < 0.05. a indicates p < 0.05 for spleen vs. lung, kidney, intestine, and pancreas; b indicates p < 0.05 for liver vs. pancreas.
